# Thyroid transcription factor‐1 expression in lung neuroendocrine tumours: a gender-related biomarker?

**DOI:** 10.1007/s12020-023-03542-0

**Published:** 2023-09-30

**Authors:** Anna La Salvia, Alessandra Siciliani, Maria Rinzivillo, Monica Verrico, Roberto Baldelli, Giulia Puliani, Roberta Modica, Isabella Zanata, Irene Persano, Giuseppe Fanciulli, Massimiliano Bassi, Massimiliano Mancini, Stefania Bellino, Elisa Giannetta, Mohsen Ibrahim, Francesco Panzuto, Maria Pia Brizzi, Antongiulio Faggiano

**Affiliations:** 1 National Center for Drug Research and Evaluation, National Institute of Health (ISS), Rome, Italy; 2 Department of Thoracic Surgery, Sant’Andrea University Hospital, Rome, Italy; 3 Digestive Disease Unit, ENETS Center of Excellence, Sant’Andrea University Hospital, Rome, Italy; 4 Department of Radiological, Oncological, and Pathological Sciences, Sapienza University of Rome, Rome, Italy; 5 Endocrinology Unit, Department of Oncology and Medical Specialities, A.O. San Camillo-Forlanini, Rome, Italy; 6 Oncological Endocrinology Unit, IRCCS Regina Elena National Cancer Institute, Rome, Italy; 7 Department of Clinical Medicine and Surgery, University of Naples Federico II, Naples, Italy; 8 Department of Medical Sciences, Section of Endocrinology and Internal Medicine, University of Ferrara, Ferrara, Italy; 9 Department of Oncology, A.O.U. San Luigi Gonzaga Hospital, Orbassano, TO Italy; 10 Department of Medicine, Surgery and Pharmacy, University of Sassari, Sassari, Italy; 11 Endocrine Oncology Program, Endocrine Unit, Azienda Ospedaliero-Universitaria (AOU) Sassari, Sassari, Italy; 12 Department of Thoracic Surgery, Policlinico Umberto I, “Sapienza” University of Rome, Rome, Italy; 13 Division of Morphologic and Molecular Pathology Unit, S. Andrea Hospital, Rome, Italy; 14 Department of Experimental Medicine, “Sapienza” University of Rome, Rome, Italy; 15 Department of Medical-Surgical Sciences and Translational Medicine, Sapienza University of Rome, Rome, Italy; 16 Endocrinology Unit, Department of Clinical and Molecular Medicine, Sant’Andrea Hospital, Sapienza University of Rome, ENETS Center of Excellence, Rome, Italy

**Keywords:** Lung neuroendocrine tumours, Thyroid transcription factor‐1 (TTF-1), Biomarkers, Gender medicine

## Abstract

**Purpose:**

Thyroid transcription factor‐1 (TTF‐1) assessed by immunohistochemistry (IHC) is a specific biomarker for lung adenocarcinoma, and is commonly used to confirm the pulmonary origin of neuroendocrine tumours (NET). The majority of the available data suggest that TTF-1 is favourable prognostic biomarker for lung adenocarcinomas, whereas its role is more conflicting for lung NET. The main aim of this multicenter retrospective study was to investigate the potentially relevant associations between TTF-1 biomarker and clinical and pathological features of the study population, as well as determine TTF-1 prognostic effect on the clinical outcome of the patients.

**Methods:**

A multicentre retrospective study was conducted on 155 surgically-removed lung NET, with available IHC TTF-1 assessment.

**Results:**

Median age was 59.5 years (range 13–86), 97 patients (62.6%) were females, 31 cases (20%) were atypical carcinoids, 4 (2.6%) had TNM stage IV. Mitotic count ≥2 per 10 high-power field was found in 35 (22.6%) subjects, whereas necrosis was detected in 20 patients (12.9%). TTF-1 was positive in 78 cases (50.3%). The median overall survival was 46.9 months (range 0.6–323) and the median progression-free survival was 39.1 months (range 0.6–323). Statistically significant associations were found between (1) TTF-1 positivity and female sex (*p* = 0.007); and among (2) TTF-1 positivity and the absence of necrosis (*p* = 0.018).

**Conclusions:**

This study highlights that TTF-1 positivity differs according to sex in lung NET, with a more common TTF-1 positive staining in female. Moreover, TTF-1 positivity correlated with the absence of necrosis. These data suggest that TTF-1 could potentially represent a gender-related biomarker for lung NET.

## Introduction

Thyroid transcription factor‐1 (TTF‐1) is a 38‐kD transcription factor that is normally expressed in adult thyroid and lung tissue [1, 2], with a relevant role in both thyroid and lung differentiation, development, and functional maintenance [3]. In the context of lung cancer, specifically non-small cell lung carcinoma (NSCLC), TTF-1 is expressed in nearly 75% of lung adenocarcinoma, whereas lung squamous cell carcinoma does not express TTF-1. Thereby TTF‐1, assessed by immunohistochemistry (IHC), is considered a specific diagnostic biomarker for lung adenocarcinoma [4]. In addition, in the neuroendocrine tumours (NET) setting, TTF-1 is commonly used to confirm the pulmonary origin of the tumour [5, 6], both for typical carcinoid, TC, as well as for atypical carcinoid, AC.

Available evidences suggest a specificity of this biomarker for the differential diagnosis of NET from different primary sites, but at the same time highlight that TTF-1 presents an extremely heterogeneous expression in lung NET [7, 8]. Several studies have evaluated the prognostic role of TTF-1 in NSCLC [9], suggesting that TTF-1 is favourable prognostic biomarker for lung adenocarcinomas [10]. Unfortunately, its role is more conflicting for lung NET [11, 12]. This issue is of note, in the setting of lung NET, given that established prognostic factors, beyond the histological subtype of TC (associated to a better prognosis) vs. AC and the pathological TNM stage, are missing. In this context, in a previous work by our group we postulated a role for primary lung NET laterality [13]. In that retrospective multicentre analysis, tumours located in the left lung clearly presented a higher biological aggressiveness (expressed as higher proliferation index, as mitotic count and Ki-67, more common presence of necrosis and higher tumour grade). A clinical impact of these differences at the pathological level was further confirmed by univariable analysis and Cox regression-based multivariable model. In addition, we demonstrated different angiogenic pattern, in terms of micro vessel density (MVD) by CD34 immunohistochemical (IHC) staining and hypoxia, according to lung NET laterality [14], with right tumours presenting higher angiogenesis rates, and left tumours associated more commonly with hypoxia. In this study, TTF-1-negative cases presented a lower OS, with a 1-year OS rate of 90.0% vs. 100% for TTF-1-positive cases. Notably, the difference resulted as more evident when comparing 10-years OS rates, with 60% for TTF-1 negative cases vs. 100% for TTF-1 positive cases. However, the impact on OS was not statistically significant in the Cox regression analysis (*p* = 0.468, HR: 0.14, 95% CI 0.001–1414.981) [14], confirming the controversial literature evidences about a possible prognostic role of TTF-1 for lung NET.

Therefore, the main aim of this multicentre and retrospective study was to evaluate the potential value of TTF-1 for lung NET, assessing its potentially relevant associations with key clinical and pathological variables and evaluating its prognostic role in a selected and homogeneous population of surgically resected lung NET.

## Materials and methods

A multicentre retrospective study was performed including patients with a confirmed histological diagnosis of lung NET classified as TC (NET G1) or AC (NET G2) according to WHO 2022 classification [15], who were diagnosed at the study Institutions. The resected lung tissues were fixed in a 10% neutral buffered formalin solution and the specimens were set in paraffin and sliced (2-µm-thick for each section). Each included case was stained with haematoxylin–eosin, chromogranin A, Synaptophysin, TTF-1 and Ki-67. IHC marker expression was quantified by expression intensity (weak, moderate, strong) and the percentage of IHC marker-positive tumour cells in fields of view of 200-fold magnification by experienced pathologist of each centre. Chromogranin A and synaptophysin were considered positive if >90% of the neoplastic cells exhibited at least moderate staining intensity. TTF-1 was scored positive if >10% of the nuclei of tumour cells were positive and the staining intensity was moderate or strong. The Ki-67 index was obtained by counting the positive tumour cells in areas of higher nuclear labelling (so-called hotspots) and was expressed as a percentage.

Inclusion criteria, beyond the diagnosis, were: (1) the surgical removal of the primary tumour, (2) patients with available IHC assessment for TTF-1. We chose these criteria to (1) ensure a homogeneous population and, also, comparable tumoral samples for histopathological evaluation, (2) study specifically the TTF-1 significance as biomarker for lung NET, given the conflicting data available in literature. After patients’ selection, we carefully collected their relevant clinical and pathological data in a dedicated database.

Descriptive statistical analyses were performed on the overall collected data. Univariable analysis by Chi-square test and multivariable analysis using a logistic regression model were applied to investigate the association among TTF-1 expression and demographic and clinical factors considered relevant for lung NET (i.e. sex, age, smoking history, disease stage, tumour grade, Ki-67 value and tumour subtype). Univariable and multivariable Cox regression analysis was performed to evaluate the prognostic role of TTF-1 on the patients’ outcome (i.e. progression-free survival and overall survival) together with other relevant factors., All statistical analyses were performed using IBM-SPSS version 25 (IBM Corporation, New York, United States of America). *p* values < 0.05 were considered statistically significant.

## Results

### Study population

Patients’ characteristics are summarised in Table 1.

**Table 1:** Clinico-pathological characteristics of the study population

Characteristic	*N* = 155 (100%)
Sex	
Male	58 (37.4%)
Female	97 (62.6%)
Median age	
	59.5 years (13–86)
Smoke	
Yes	40 (25.8%)
No	79 (51.0%)
NA	36 (23.2%)
Tumour location	
Peripheral	69 (44.5%)
Central	85 (54.8%)
NA	1 (0.6%)
Tumour side (lung parenchyma)	
Left	66 (42.6%)
Right	89 (57.4%)
Diagnosis	
Typical carcinoid	124 (80.0%)
Atypical carcinoid	31 (20.0%)
Stage at the diagnosis	
I	96 (61.9%)
II	28 (18.1%)
III	15 (9.7%)
IV	4 (2.6%)
NA	12 (7.7%)
T	
T1	89 (57.4%)
T2	44 (28.4%)
T3	8 (5.2%)
T4	5 (3.2%)
NA	9 (5.8%)
Nodal status	
N0	114 (73.5%)
N+	31 (20.0%)
NA	10 (6.5%)
18-FDG PET positivity	
Yes	67 (43.2%)
No	34 (21.9%)
NA	54 (34.8%)
68-Gallium PET/Octreoscan positivity	
Yes	24 (15.5%)
No	12 (7.7%)
NA	119 (76.8%)
Mitosis	
<2 per 10 HPF	114 (73.5%)
≥2 per 10 HPF	35 (22.6%)
NA	6 (3.9%)
Necrosis	
Yes	20 (12.9%)
No	133 (85.8%)
NA	2 (1.3%)
Ki67 (%)	
1–2	100 (64.5%)
3–19	42 (27.1%)
>20	5 (3.2%)
NA	8 (5.2%)
Grade	
G1	103 (66.5%)
G2	40 (25.8%)
G3	5 (3.2%)
NA	7 (4.5%)
Synaptophysin	
Positive	137 (88.4%)
Negative	8 (5.2%)
NA	10 (6.5%)
Chromogranin A	
Positive	139 (89.7%)
Negative	11 (7.1%)
NA	5 (3.2%)
TTF-1	
Positive	78 (50.3%)
Negative	77 (49.7%)
Type of surgery	
Pneumonectomy	9 (5.8%)
Bilobectomy	5 (3.2%)
Lobectomy	91 (58.7%)
Sleeve resection	4 (2.6%)
Segmental resection	10 (6.5%)
Wedge resection	14 (9.0%)
Other	6 (3.9%)
NA	16 (10.3%)
Progression	
Yes	38 (24.5%)
No	117 (75.5%)
Alive	
Yes	128 (82.6%)
No	14 (9.0%)
NA	13 (8.4%)
Median OS	
	46.9 months (0.6–323)
Median PFS	
	39.1 months (0.6–323)

*HPF* high-power field, *NA* not available, *OS* overall survival, *PFS* progression-free survival

Overall, we included 155 surgically removed lung NET. Median age was 59.5 years (13–86), *N* = 97 (62.6%) were females, *N* = 40 (25.8%) were smokers, *N* = 31 (20%) were ACs, *N* = 85 (54.8%) were centrally located, *N* = 89 (57.4%) were located in the right lung, *N* = 4 patients (2.6%) presented a TNM stage IV at the diagnosis. Mitotic count was ≥2 per 10 high-power field (HPF) in *N* = 35 cases (22.6%), necrosis was present in *N* = 20 (12.9%). Grade 1 was reported in 103 cases (66.5%), whereas Ki-67 was >20% in *N* = 5 (3.2%). TTF-1 was positive in 78 cases (50.3%), Chromogranin A in 139 (89.7%) and Synaptophysin in 137 (88.4%), respectively. The most common type of surgery was lobectomy in 91 patients (58.7%). Median overall survival (OS) was 46.9 months (0.6–323), median progression-free survival (PFS) was 39.1 months (0.6–323).

**Fig. 1:**
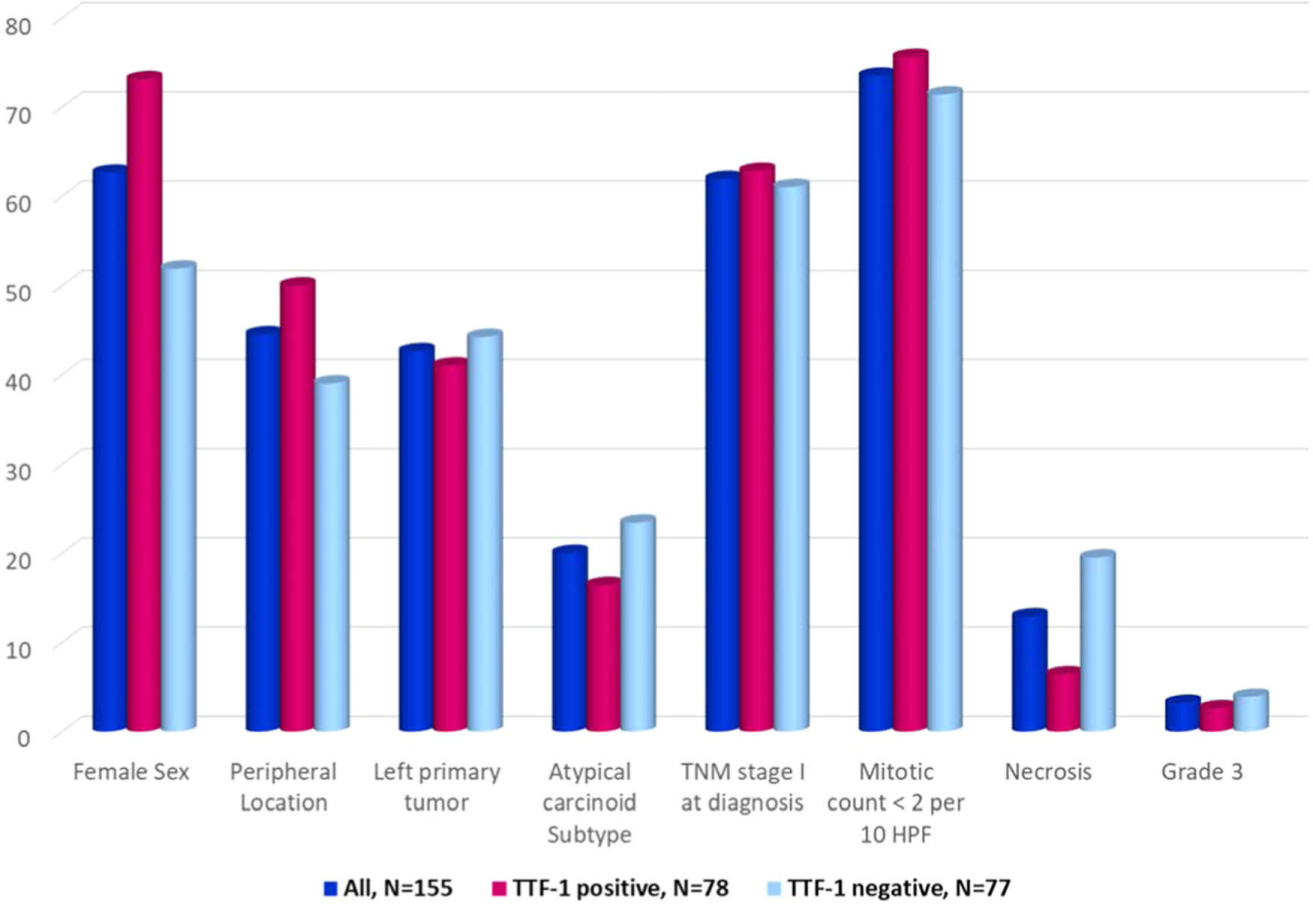
Key features: all vs. TTF-1 positive cases. In this graph are compared the distributions of all lung NET included in the study vs. TTF-1 positive cases in selected clinical and pathological variables (as sex, central vs. peripheral location of the primary tumour, tumour laterality, histopathological subtype of TC vs. AC, TNM stage, mitotic count, presence of necrosis and tumour grade G3 vs. G1 and G2). Significant (*p* < 0.05) associations are marked with an asterisk (*)

Specifically, as detailed above, we focused on TTF-1 positive cases. Comparison among the prevalence and distribution of selected clinical and pathological features among the entire study population and the subgroup of TTF-1 positive patients are reported in Fig. 1. All the collected data of TTF-1 positive cases are detailed in Supplementary Material 1.

### Associations

**Fig. 2:**
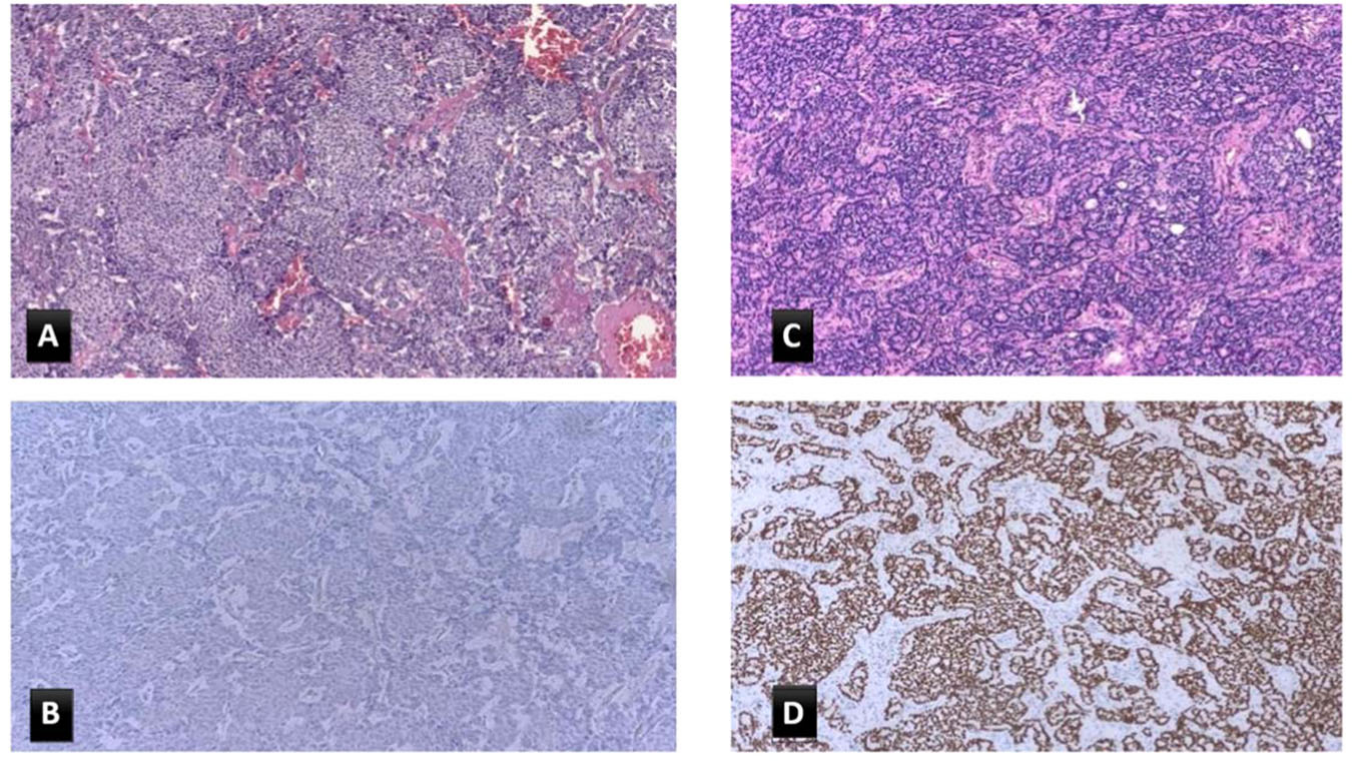
Two exemplificative cases of lung NET comparing TTF-1 expression according to sex. Male patient (**A**, **B**) shows a well differentiated morphological pattern with the absence of necrosis, TC (**A**) with negative staining for TTF-1 (**B**). Female patient (**C**, **D**) with diagnosis of TC (**C**) but positive TTF-1 IHC staining (**D**). **A**, **C** Haematoxylin and eosin stain,10X, and (**B**, **D**) TTF-1 immunohistochemical stain, 10X

Statistically significant associations were found between (1) TTF-1 positivity and female sex (*p* = 0.007); and among (2) TTF-1 positivity and the absence of necrosis (*p* = 0.018). No other relevant associations were detected between TTF-1 and the remaining clinical or pathological variables. Notably, female sex correlates with the absence of necrosis (*p* = 0.002). Results of the multivariable analysis by the logistic regression model to take into account other potentially confounding variables confirmed the association between TTF-1 positivity and female sex (*p* = 0.042) (Table 2). Two exemplificative cases of TC, one female with TTF-1 positive tumour and a male with TTF-1 negative tumour, are depicted in Fig. 2.

**Table 2:** Logistic regression model: adjusted odds ratios of TTF-1 expression

Multivariable analysis	OR	95% CI		*p* value
Gender (female vs. male)	3.90	1.05	14.46	0.042
Age (lower vs. higher median value)	1.39	0.38	5.05	0.618
Smoke (no vs. yes)	5.42	1.09	26.88	0.039
Stage (I vs. II–III–IV)	1.67	0.04	61.80	0.536
Grade (G3 vs. G1–G2)	0.31	0.00	22.11	0.590
Ki-67 index	0.62	0.04	9.55	0.730
Histological subtype (TC vs. AC)	0.44	0.14	1.38	0.159

*OR* odds ratio, *CI* confidence interval

Of note, any difference in TTF-1 expression according to the laterality of the primary tumour was observed. TTF-1 positive cases were equally distributed in right and left lung (TTF-1 positive lung NET: 46 right-located and 32 left-located; TTF-1 negative tumours: 43 right-located and 34 left-located; *p* = 0.694).

### Impact on survival

Although the statistical significance was not reached (*p* = 0.609 for OS and *p* = 0.368 for PFS), a favourable trend in OS and PFS was found for TTF-1 positivity, with an estimated OS of 126 months for TTF-1 negative cases and 200 months for positive cases; in terms of PFS the estimated months for TTF-1 negative cases was 107 vs. 169 for positive ones. In the multivariable analysis, tumour stage emerged as the unique factor with independent prognostic impact (*p* = 0.004) on OS (Table 3); however, a trend for TTF-1 expression, age, and histological subtype effects was detected.

**Table 3:** Cox regression model: adjusted hazard ratios of overall survival

Multivariable analysis	HR	95% CI		*p* value
TTF-1 (positive vs. negative)	0.06	0.00	1.31	0.073
Gender (Female vs. Male)	1.66	0.10	28.98	0.727
Age (lower vs. higher median value)	0.10	0.01	1.14	0.064
Smoke (no vs. yes)	0.86	0.09	8.59	0.898
Stage (I vs. II–III–IV)	0.00	0.00	0.08	0.004
Grade (G3 vs. G1–G2)	2.07	0.15	28.20	0.585
Histological subtype (TC vs. AC)	0.02	0.00	1.07	0.054

*HR* hazard ratio, *CI* confidence interval

## Discussion

Unambiguous evidence supports the relevance of gender difference in oncology, from the diagnosis to the response to treatments and treatments’ side-effects prolife [16–18]. Overall, sex significantly influences the clinical and pathological features of cancer patients. These include disparities in incidence and mortality rates, clinical presentations including age, screening participation rates, site, stage and treatment utilisation, histopathology (including genetic and molecular features) and survival [19–21]. Environmental and behavioural factors (e.g. smoking habit or metabolic syndrome onset) play a key role in this context [22–24]. Moreover, biological (e.g. sex hormones) features have been showed to contribute to the differential risk in several tumour types [25, 26]. In the field of NET, indeed, the majority of available data arise from studies on gastro-entero-pancreatic (GEP) neuroendocrine neoplasms (NEN) [27]. A large population study, including 15,202 patients with pancreatic neuroendocrine tumours from The National Cancer Database (NCDB), suggested that men had more frequently tumours >2 cm, and poorly or undifferentiated tumours if compared to women [28]. Notably, no significant differences were found in the rates of lymph node involvement and metastatic recurrence after the surgical removal of the primary tumour. At the molecular level, MEN1 and DAXX mutations resulted more common in males and TP53 mutations in females, respectively. However, these data lacked to be confirmed at the multivariable analysis. Data of the Surveillance, Epidemiology and End Results Research (SEER) registry, based on 43,751 patients with GEP-NETs, demonstrated with multivariable analyses a prognostic value (in terms of OS) for sex with women associated to better outcomes (*p* < 0.001) [29]. In this analysis the 3-year survival rates resulted 84.6% and 87.7% and the 5-years survival rates of 80% vs. 84% for male and female, respectively. For lung NET a sex-difference has been described, with, even in this case, more favourable trends in female in a previous work by our group [13]. According to the Cox-univariate regression model a significant impact on patients’ outcome for sex was demonstrated, with male sex associated with dismal PFS (*p* < 0.0001) and OS (*p* < 0.0001). These data have been further confirmed by data coming from SEER registry, where female sex was associated with better OS compared with male sex *(p* < 0.001*)* [30]. Furthermore, few works suggest biological differences in terms of tumour aggressiveness in relation to patients’ sex [13]. Female sex has been associated with a more indolent disease, both considering a lower tumour stage (specifically, negative nodal status vs. positive) and also with regards to pathological features, as lower tumour grade (G1-2 vs. G3), lower Ki67 index and reduced mitotic count. In addition, a sex imbalance of the histological subtype (TC vs. AC) in males and females has been reported [31]. However, a sex-related distribution of the main IHC NET biomarkers (Chromogranin A, NSE and, specifically, TTF-1) has never been reported in literature. A retrospective study including 11 carcinoid tumorlets (TLs), 36 TC, 17 AC and 16 large cell neuroendocrine carcinomas (LCNECs) showed a more common positive TTF-1 IHC staining in LCNECs (5 of 6 positive cases), followed by TLs (4 of 8) respect to AC (1 of 4), and TC (0 of 10) [32]. Interestingly, in this study the percentage of female was higher in these two categories, LCNEC and TLs, whereas both TC and AC were well-balanced among male and female. In the current work we observed a statistically significant association between the IHC positivity for TTF-1 and female lung NET patients *(p* = 0.007*)*. This result deserves further studies to confirm a potential biological significance of TTF-1 expression in connection with the sex of lung NET patients, with potentially relevant implications in the diagnostic work-up of these tumours.

TTF-1^+^ alveolar type II epithelial cells have been demonstrated to be the major source of vascular endothelial growth factor (VEGF) in the lung [33, 34]. At the molecular level, TTF-1 has been postulated to positively regulate VEGF expression and the major signalling receptor for VEGF as VEGFR2 lung cancer epithelial cells [34, 35]. TTF-1, indeed, has been suggested to reprogramme lung cancer secreted proteome into an antiangiogenic state. Interestingly, TTF-1 has been assessed as a potential predictive factor for antiangiogenic treatment in non-squamous NSCLC [36]. In this study, the 92 TTF-1-positive patients presented higher response rates (51.4% vs. 27.3%, *p* = 0.027) and PFS (216 days vs. 137 days, *p* = 0.012) in the group treated with the antiangiogenic bevacizumab to standard chemotherapy, whereas in TTF-1-negative patients no clinical benefit was obtained by the combination therapy (chemotherapy plus bevacizumab). Unfortunately, data about VEGF expression and TTF-1 in lung NET are lacking. In a previous work by our group, we demonstrated a significant association between the absence of expression of the TTF-1 and the presence of hypoxia (in 14/16, 87.5%, of TTF-1-negative cases, *p* = 0.012). Among hypoxia-negative cases, 11/13 (84.6%) were TTF-1 positive, whereas among hypoxia-positive cases, 10/24 expressed TTF-1 [14]. In the present study we detected a statistically significant correlation among TTF-1 positivity and the absence of necrosis (*p* = 0.018). Taken all together this data, it is possible to hypothesise that TTF-1 may be positively linked to increased angiogenesis, and associated with lower hypoxia and the absence of necrosis in lung neoplasms, potentially including lung NET.

Finally, in the present work we investigated the prognostic value of TTF-1 in our lung NET population. According to available evidences, TTF-1 positivity is considered an established positive prognostic factor for lung adenocarcinomas [10]. In lung NET field, more conflicting data have been reported. In a retrospective series of 370 lung NET, a difference in IHC positivity for TTF‐1 was found between patients with higher or lower Ki-67 [11]. Overall, a positive staining for TTF-1 was detected in 49 (17.1%) of the included lung NET, with TTF‐1 positivity in 30 (13.0%) of the low Ki‐67 group of patients and in 19 (34.5%) cases of the high Ki‐67 group. The second group (with higher Ki-67) was associated to a worse prognosis (*p* < 0.0001). Also, TTF-1 positivity correlated with a reduced survival outcome (*p* = 0.03). In a retrospective study of 34 lung NET treated with peptide receptor radionuclide therapy with (177) Lu-DOTATATE (Lu-PRRT), survival outcomes in terms of PFS were better in TTF-1 negative cases if compared to TTF-1 positive ones (26.3 vs. 7.2 months, respectively, *p* = 0.0009) [37]. However, these data have not been confirmed in subsequent works [12, 38]. A retrospective analysis of 108 lung NET lacked to demonstrate a correlation between TTF-1 positivity and patient outcomes [12,]. In another study, TTF-1 was positive in 78% of the 133 lung NET cases but was not associated with patients’ survival [38]. In the present study, in line with the available literature data, no significant association among TTF-1 expression and patient survival was observed (according to univariable and also multivariable model), despite a favourable trend for TTF-1 positive cases was noticed. Further prospective studies are encouraged to determine if this biomarker has a prognostic relevance for lung NET.

## Conclusions

TTF-1 is a well-known biomarker for lung neoplasms, above all for adenocarcinomas. For lung NET, the diagnostic value of is TTF-1 debated, despite its widespread use in the clinical practice. TTF-1’s role as a prognostic biomarker for lung NET is still uncertain, and also in our analysis a not significant trend has been detected. TTF-1 negativity resulted associated with the presence of necrosis, in analogy with the few available literature data that have showed a higher expression of TTF-1 in conditions of higher angiogenesis and lower hypoxia. Notably, our study provides, to the best of our knowledge, the first evidence of a correlation among TTF-1 positivity and female sex, suggesting a potential biological and clinical relevance of this observation. Further studies with larger and independent patients’ populations are needed to confirm TTF-1 role as a gender-related biomarker for lung NET.

### Supplementary information

Supplementary Material 1

## Data Availability

All data generated or analysed during this study are included in this article. Further enquiries can be directed to the corresponding author.
