# Supplementary material for: Thyroid transcription factor‐1 expression in lung neuroendocrine tumours: a gender-related biomarker?
Source: Endocrine. 2023 Sep 30;83(2):519–26. doi: 10.1007/s12020-023-03542-0 (PMC10850191; doi:10.1007/s12020-023-03542-0)
Supplement: Supplementary file 1 — Supplementary Material 1 [file 12020_2023_3542_MOESM1_ESM.docx]

| Supplementary Material 1. Key clinico-pathological featuress of TTF-1 positive cases | |
| --- | --- |
| Characteristic | **N=78 (100%)** |
| Sex |  |
| Male | 21 (26.9%) |
| Female | 57 (73.1%) |
| Median age |  |
|  | 61.5 years (22–86) |
| Smoke |  |
| Yes | 20 (25.6%) |
| No | 43 (55.1%) |
| NA | 15 (19.2%) |
| Tumor location |  |
| Peripheral | 39 (50.0%) |
| Central | 39 (50.0%) |
| Tumor side (lung parenchyma) |  |
| Left | 32 (41.0%) |
| Right | 46 (59.0%) |
| Diagnosis |  |
| Typical carcinoid | 65 (83.3%) |
| Atypical carcinoid | 13 (16.7%) |
| Stage at the diagnosis |  |
| I | 49 (62.8%) |
| II | 17 (21.8%) |
| III | 5 (6.4%) |
| IV | 2 (2.6%) |
| NA | 5 (6.4%) |
| T |  |
| T1 | 49 (62.8%) |
| T2 | 20 (25.6%) |
| T3 | 3 (3.8%) |
| T4 | 2 (2.6%) |
| NA | 4 (5.1%) |
| Nodal status |  |
| N0 | 57 (73.1%) |
| N+  NA | 15 (19.2%)  6 (7.7%) |
| 18-FDG PET positivity |  |
| Yes | 37 (47.4%) |
| No | 16 (20.5%) |
| NA | 25 (32.1%) |
| 68-Gallium PET/Octreoscan positivity |  |
| Yes | 13 (16.7%) |
| No | 9 (11.5%) |
| NA | 56 (71.8%) |
| Mitosis |  |
| < 2 per 10 HPF | 59 (75.6%) |
| ≥ 2 per 10 HPF | 16 (20.5%) |
| NA | 3 (3.8%) |
| Necrosis |  |
| Yes | 5 (6.4%) |
| No | 71 (91.0%) |
| NA | 2 (2.6%) |
| Ki67 (%) |  |
| 1-2 | 50 (64.1%) |
| 3-19 | 20 (25.6%) |
| >20 | 2 (2.6%) |
| NA | 6 (7.7%) |
| Grade |  |
| G1 | 53 (67.9%) |
| G2 | 19 (24.4%) |
| G3 | 2 (2.6%) |
| NA | 4 (5.1%) |
| Synaptophysin |  |
| Positive | 68 (87.2%) |
| Negative | 3 (3.8%) |
| NA | 7 (9.0%) |
| Chromogranin A |  |
| Positive | 72 (92.3%) |
| Negative | 4 (5.1%) |
| NA | 2 (2.6%) |
| Type of surgery |  |
| Pneumonectomy | 4 (5.1%) |
| Bilobectomy | 3 (3.8%) |
| Lobectomy | 45 (57.7%) |
| Segmental Resection | 6 (7.7%) |
| Wedge Resection | 6 (7.7%) |
| Other | 4 (5.1%) |
| NA | 10 (12.8%) |
| Progression |  |
| Yes | 16 (20.5%) |
| No | 62 (79.5%) |
| Alive |  |
| Yes | 65 (83.3%) |
| No | 6 (7.7%) |
| NA | 7 (9.0%) |
| Median OS |  |
|  | 30 months (0.6–323) |
| Median PFS |  |
|  | 24 months (0.6–323) |

*Abbreviations: HPF: high power field, NA: not available, OS: overall survival, PFS: progression free survival.*
